# A comparative study of next-generation sequencing and fragment analysis for the detection and allelic ratio determination of *FLT3* internal tandem duplication

**DOI:** 10.1186/s13000-022-01202-x

**Published:** 2022-01-26

**Authors:** Jin Ju Kim, Kwang Seob Lee, Taek Gyu Lee, Seungjae Lee, Saeam Shin, Seung-Tae Lee

**Affiliations:** 1 Department of Laboratory Medicine, Yonsei University College of Medicine, Severance Hospital, 50-1 Yonsei-ro, Seodaemun-gu, Seoul, 03722 Republic of Korea; 2 Brain Korea 21 PLUS Project for Medical Science, Yonsei University, Seoul, Republic of Korea

**Keywords:** Acute myeloid leukemia, *FLT3* internal tandem duplication, Fragment analysis, Allele ratio

## Abstract

**Background:**

Currently, *FLT3* internal tandem duplication (ITD) is tested by fragment analysis. With next-generation sequencing (NGS), however, not only *FLT3* ITD but also other mutations can be detected, which can provide more genetic information on disease.

**Methods:**

We retrospectively reviewed the results of two tests—fragment analysis and a custom-designed, hybridization capture-based, targeted NGS panel—performed simultaneously. We used the Pindel algorithm to detect *FLT3* ITD mutations.

**Results:**

Among 277 bone marrow aspirate samples tested by NGS and fragment analysis, the results revealed 99.6% concordance in *FLT3* ITD detection. Overall, the allele frequency (AF) attained by NGS positively correlated with the standard allelic ratio (AR) attained by fragment analysis, with a Spearman correlation coefficient (r) of 0.757 (95% confidence interval: 0.627–0.846; *p* < 0.001). It was concluded that an AF of 0.11 attained by NGS is the most appropriate cutoff value (with 85.3% sensitivity and 86.7% specificity) for high mutation burden criterion presented by guidelines.

**Conclusion:**

Sensitive *FLT3* ITD detection with comprehensive information of other mutation offered by NGS could be a useful tool in clinical laboratories. Future studies will be needed to evaluate and standardize NGS AF cutoff to predict actual clinical outcomes.

**Supplementary Information:**

The online version contains supplementary material available at 10.1186/s13000-022-01202-x.

## Introduction

Internal tandem duplication (ITD) in the Fms-related receptor tyrosine kinase 3 (*FLT3*) gene is found in approximately 20 to 30% of cases of normal karyotype acute myeloid leukemia (AML) [1]. *FLT3* ITD is a driver of leukemogenesis, resulting in constitutive activation of the FLT3 receptor and autonomous proliferation of cells [2]. It is transcribed in-frame and occurs in from three to a few hundred base pairs commonly in the juxtamembrane domain region [3]. Patients with *FLT3* ITD are associated with a poor prognosis due to a high risk of recurrence and shorter overall survival [1]. Recently, two *FLT3* tyrosine kinase inhibitors, midostaurin and gilteritinib, were approved and are used in patients with *FLT3*-mutated AML [4, 5].

Polymerase chain reaction (PCR) with capillary electrophoresis-based DNA fragment analyses is the current standard method for the detection and quantification of *FLT3* ITD [6–8]. Clinical guidelines, such as the European LeukemiaNet (ELN) and National Comprehensive Cancer Network consensus (NCCN) guidelines, define a *FLT3* ITD allelic ratio cutoff of 0.5 as measured by fragment analysis as the optimal risk-stratification criterion in AML [6, 9].

In the last few years, many molecular diagnostic laboratories have come to employ next-generation sequencing (NGS), which has the advantage of identifying AML-related somatic mutations in multiple genes. Quantitative analysis is also possible using NGS; however, the detection and quantification of large insertion/deletion (indel) variants are problematic.

In this work, we retrospectively analyzed the clinical data of patients who underwent *FLT3* ITD tests using fragment analysis and NGS applied at the primary source to assess the correlation between these two methods of mutation detection and burden measurement.

## Materials and methods

### Patients and samples

This study protocol was approved by the Institutional Review Board of Yonsei University Severance Hospital (approval no. 2021–0389-001). We retrospectively reviewed the concurrent test results of fragment analysis for *FLT3* ITD and a targeted NGS panel deployed in our laboratory from January 2017 to March 2021. Patients’ diagnosis and response criteria were followed with the 2016 World Health Organization classification of myeloid neoplasms and acute leukemia and 2017 ELN recommendations [6, 10]. Samples were referred to the laboratory at diagnosis and follow-up for hematologic neoplasms. For both fragment analysis and NGS, genomic DNA was extracted from bone marrow or whole-blood samples using the QIAamp DNA Blood Mini Kit (Qiagen, Venlo, The Netherlands).

### Capillary electrophoretic fragment analysis

PCR was performed on genomic DNA using an AccuPower HotStart PCR Premix (Bioneer, Daejeon, Korea) and specific primers [11] (forward: 5′-FAM- TGCCTATTCCTAACTGACTCATCA--3′, reverse:5′- TCTTTGTTGCTGTCCTTCCA − 3′) to amplify *FLT3* exon 14 and 15 regions with following conditions: 94 °C for 5 min followed by 35 cycles of 94 °C for 30 s, 60 °C for 30 s and 72 °C for 60 s, and final extension at 72 °C for 7 min. Thereafter, 10 μL of the mixture of 0.5 μL fragment-length standard (GeneScan-600 LIZ Size Standard, Thermo Fisher Scientific, Foster City CA, USA) and 10 μL HiDi formamide (Thermo Fisher Scientific) was added to 1 μL of 30 times diluted PCR product. After the initial denaturation of this mixture at 95 °C, size-separation by capillary electrophoresis was performed on a 3130 Genetic Analyzer (Thermo Fisher Scientific) using the separation matrix POP-7 polymer (Thermo Fisher Scientific). Data analysis was performed using the GeneMapper version 3.2 software program (Thermo Fisher Scientific). The *FLT3* ITD allelic ratio (AR) is calculated as the ratio of the peak height of the mutant product to the peak height of the wild-type product. The detection limit of PCR-based capillary electrophoresis fragment analysis used in this study was AR of 0.01. A manual review of the *FLT3* ITD region was performed in all cases.

### Targeted NGS

NGS was performed with custom probes targeting 497 genes related to hematologic neoplasms (Supplementary Table 1). The DNA probe was used, and the *FLT3* ITD region (*FLT3* exon 14, exon 15, and intron 14) was tiled with 2× density. Prepared libraries were then hybridized with probes and sequenced on the NextSeq 550Dx instrument (Illumina, San Diego, CA, USA). The Burrows–Wheeler alignment tool was used for sequence alignment. The Pindel algorithm, which was implemented in the DxSeq Analyzer (Dxome, Seoul, Korea), was used to detect *FLT3* ITD mutations. The bioinformatics pipelines used the analysis was presented in Supplementary Fig. 1. The *FLT3* ITD allelic frequency (AF) was calculated from the number of sequencing reads showing internal tandem duplication versus the total sequence.

### Statistical analysis

The statistical analysis was performed using MedCalc version 18.2.1 (MedCalc Software, Mariakerke, Belgium). Continuous variables are described using mean values, whereas categorical variables are presented as percentages. Concordance was measured using the percent of agreement between the two assays. A metric variable correlation between AR values obtained by fragment analysis versus AF values obtained by NGS was conducted using Passing Bablok regression analysis with Spearman correlation coefficient. A receiver-operating characteristic (ROC) analysis was performed to determine the sensitivity, specificity, and optimal diagnostic cutoff values of NGS in screening for high *FLT3* ITD burden cases. The most appropriate cutoff value was chosen according to ROC analysis, and the area under the ROC curve (AUC) was calculated. Results were considered statistically significant at the level of *p* < 0.05.

## Results

### Clinical and molecular characteristics of FLT3 ITD-positive cases identified by NGS

Among 277 samples tested NGS and fragment analysis, 260 cases were collected from initial samples of patients, including AML (*n* = 236), B-lymphoblastic leukemia (*n* = 5), myelodysplastic syndrome/cytopenia (*n* = 13), mixed-phenotype acute leukemia (*n* = 2), myeloid sarcoma (*n* = 3) and pure erythroid leukemia (*n* = 1). The remaining 17 samples were follow-up samples taken from AML patients. Among 277 samples, *FLT3* ITD was detected by both methods in 64 cases and only by NGS in one case. (Table 1). 61 initial samples with *FLT3* ITD positive by both methods were collected from AML patients, including AML with mutated *NPM1* (*n* = 24), AML with maturation (*n* = 14), AML with myelodysplasia-related change (*n* = 5), acute myelomonocytic leukemia (*n* = 5), AML with biallelic mutation of *CEBPA* (*n* = 3), acute promyelocytic leukemia (*n* = 3), AML with mutated *RUNX1* (*n* = 2), AML with t (8;21)(q22;q22.1) (*n* = 2), acute monocytic leukemia (*n* = 2) and AML with inv. (16)(p13.1q22) (*n* = 1) (Supplementary Table 2). Aside from 3 acute promyelocytic leukemia patients and one patients without further treatment record, all patients received either standard 3 + 7 induction chemotherapy (*n* = 35) or hypomethylating agent therapy (*n* = 22). 19 out of 60 patients with treatment medical record are treated with FLT3 inhibitor.

**Table 1:** Summary of diagnosis and *FLT3* ITD results from patients

Diagnosis	Sample, ***n***	NGS		Fragment analysis	
		***FLT3*** ITD-positive cases, ***n*** (%)	Mean allelic frequency (range)	***FLT3*** ITD-positive case, ***n*** (%)	Mean allelic ratio (range)
**Initial**	**260**	**61 (23.5)**	**0.16 (0.01–0.94)**	**61 (23.5)**	**1.24 (0.02–28.34)**
AML (APL included)	236	61 (25.8)	0.16 (0.01–0.94)	61 (25.8)	1.24 (0.02–28.34)
BLL	5	0	–	0	–
MDS/cytopenia	13	0		0	
MPAL	2	0	–	0	–
Myeloid sarcoma	3	0	–	0	–
Pure erythroid leukemia	1	0	–	0	–
**Follow-up**	**17**	**4 (23.5)**	**0.26 (0.01–0.78)**	**3 (17.6)**	**4.77 (0.62–12.56)**
AML in complete remission	13	0	–	0	–
AML in relapse/residual blast	4	4 (100.0)	0.26 (0.01–0.78)	3 (75.0)	4.77 (0.62–12.56)
**Total**	**277**	**65 (23.5)**	**0.15 (0.01–0.94)**	**64 (23.1)**	**1.39 (0.02–28.34)**

Abbreviations: AML, acute myeloid leukemia; APL, acute promyelocytic leukemia; BLL, B-lymphoblastic leukemia; MDS, myelodysplastic syndrome; MPAL, mixed-phenotype acute leukemia; NGS, next-generation sequencing, ITD, internal tandem duplication

Overall, there were 56 distinct types of *FLT3* ITD identified, ranging in size from 18 up to 216 bp, with 48 (85.7%), 3 (5.4%), and 5 (8.9%) of the 56 types falling in the ranges of less than 100, 100 to 150, and greater than 150 bp, respectively. In addition, 41 (73.2%), 8 (14.3%) and 7 (12.5%) ITDs showed insertion sites at exon 14, exon 15, and intron 14, respectively. We observed simple internal tandem duplication, as well as insertion of random extra nucleotides in the 56 types of *FLT3* ITDs: 29 (51.8%) demonstrated simple tandem duplications with a size range of 18- 216 bp; and other 27 (48.2%) had insertion of extra nucleotides of unknown origin with a size range of 24-167 bp (Supplementary Table 2).

There were 24 samples from 8 patients assessed by both methods. The studied patients’ clinical and biological characteristics are shown in Supplementary Table 3. Four patients with *FLT3* ITD detected by NGS failed to respond to the chemotherapy.

### Comparison of FLT3 ITD detection by NGS and fragment analysis

NGS demonstrated 99.6% concordance (kappa value: 0.990, 95% confidence interval (CI): 0.970–1.000) with fragment analysis in *FLT3* ITD detection, with 65 NGS-positive (size range: 18–216 bp) and 212 NGS-negative cases consistently ruled as positive and negative by fragment analysis, respectively. The AR of *FLT3* ITD was estimated to be 0.02 to 28.34 per PCR fragment analysis and the AF was estimated to be 0.005 to 0.937 per NGS analysis. In the single discordant case, *FLT3* ITD mutation was not detected by fragment analysis but exhibited a low mutation burden by NGS (AF of 0.006, 0.6%).

**Fig. 1:**
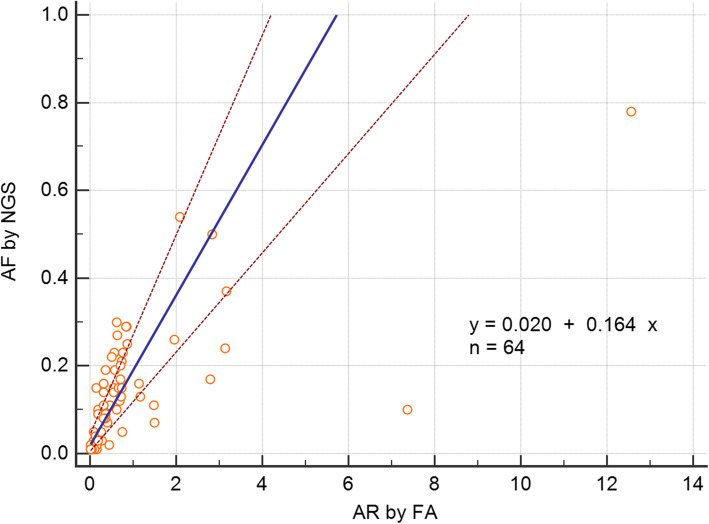
Correlation of the *FLT3* ITD mutant burden as assessed by the two study methods; Allelic ratio (AR) values from fragment analysis and allele frequency (AF) values from NGS are indicated, respectively. Abbreviations: AF, allelic frequency; NGS, next generation sequencing; AR, allelic ratio; FA, fragment analysis

Overall, the AF attained by NGS positively correlated with the AR attained by fragment analysis, with a spearman rank correlation coefficient (r) of 0.757 (95% CI: 0.627–0.846; *p* < 0.001) (Fig. 1). In comparing the tumor burden detected by the two methods, different cutoffs were applied due to the difference in burden calculation formula. A value of 0.5 as the cutoff to discern between low and high mutation burdens for fragment analysis was used according to the 2017 ELN guidelines, and a value of 0.33 for NGS was used to concord with the cutoff value by fragment analysis. The NGS results demonstrated fair agreement (kappa value: 0.14) with fragment analysis in five of 34 (14.7%) high-mutation-burden cases and 30 of 30 (100.0%) low-mutation-burden cases. Overall agreement was 54.7% (35/ 64) (Table 2).

**Table 2:** Concordance of *FLT3* ITD mutation burden classification between NGS and fragment analysis

Fragment analysis	Next-generation sequencing		Total	% Agreement
	Low mutation burden	High mutation burden		
**Low mutation burden**	30	0	30 (46.9%)	**54.7%**
**High mutation burden**	29	5	34 (53.1%)	
**Total**	**59 (92.2%)**	**5 (7.8%)**	**64**	

**Fig. 2:**
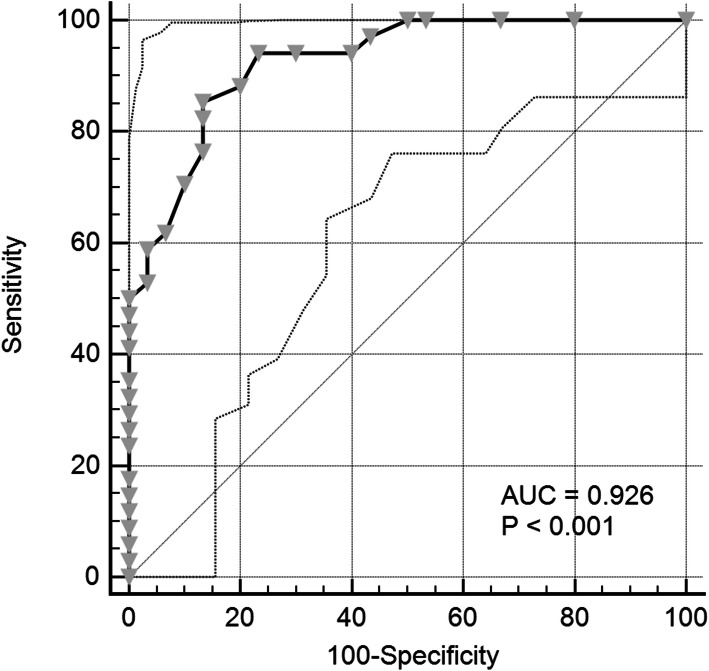
ROC curves were calculated for revealing a high *FLT3* ITD burden using AF as determined by NGS. A high *FLT3* ITD burden was indicated by an AR value of greater than 0.5 by fragment analysis. By applying the cutoff value of 0.11, the AUC was 0.926 (95% CI: 0.833–0.977). ROC curve (solid line) with 95% confidence interval (dashed lines). The dotted line is a diagonal line

We performed a ROC curve analysis to specify the appropriate cutoff value for AF per the NGS test method to determine better the degree of tumor burden adopted by the ELN guideline. Based on the results of the ROC curve analysis, the cutoff value to achieve optimal sensitivity and specificity during NGS was 0.11 (Fig. 2). If an allelic ratio of 0.11 for NGS was chosen as a new cutoff value, the sensitivity and specificity values for diagnosis were 85.3% (95% CI: 68.9–95.0%) and 86.7% (95% CI: 69.3–96.2%), respectively.

## Discussion

*FLT3* ITD mutation is a well-known predictive factor for poor prognosis in AML [12]. According to recommendations, the higher relapse rates and shorter overall survival times associated with *FLT3* ITDs are influenced by mutation burden, at the boundary of 0.5, such that a highly accurate measurement of AR is necessary [13]. Patients with *FLT3* ITD mutations carry a particularly poor prognosis such that allogeneic bone marrow transplantations during the first period of remission in this population are usually performed [14, 15]. However, relapse following transplant occurs frequently [16, 17].

Recently, FLT3 inhibitors were introduced, leading *FLT3* ITD to become an even more important indicator for determining subsequent treatment modalities [18, 19]. Minimal residual disease (MRD) studies conducted using quantitative reverse-transcription PCR have made it clear that *FLT3* ITD MRD levels following induction chemotherapy are a predictive marker of the duration of complete remission. However, sequence-specific PCR is burdensome to perform, difficult to standardize, and not routinely available outside of a research setting [20–23].

NGS enables the detection of subclinical disease in AML and allows the identification of the clonal composition and dominance of *FLT3* ITD mutations. Patients may harbor multiple *FLT3* ITD clones, and clonal allelic burden change can be monitored by NGS [20]. As the detection sensitivity of fragment analysis is known to be about 1 ~ 5% of the mutant allele burden due to PCR bias [24, 25], NGS is reported to demonstrate better analytical sensitivity than traditional fragment analysis [22, 26]. If the disease burden is lower than 1%, it may not be detectable using the fragment method but could be detected by the NGS, and this low burden of disease could be linked to relapse after intensive chemotherapy or transplantation. Moreover, with the improved detection sensitivity of NGS relative to that of fragment analysis, the lack of a need for sequence-specific probes increases the flexibility of NGS deployment [20–23].

In this study, we used hybridization and capture enrichment with Illumina sequencing to sequence *FLT3* and 496 other genes to determine the identification of *FLT3* ITD in a multigene NGS panel. We used the Pindel algorithm for the variant caller system, which is a pattern-growth algorithm using read pairs wherein one is partially or completely unaligned and is reported to be reliable for *FLT3* ITD mutation detection, thus identifying the position and length of *FLT3* ITD [21, 27]. Our results showed that the NGS method using the Pindel algorithm exhibited 99.6% concordance with fragment analysis.

**Fig. 3:**
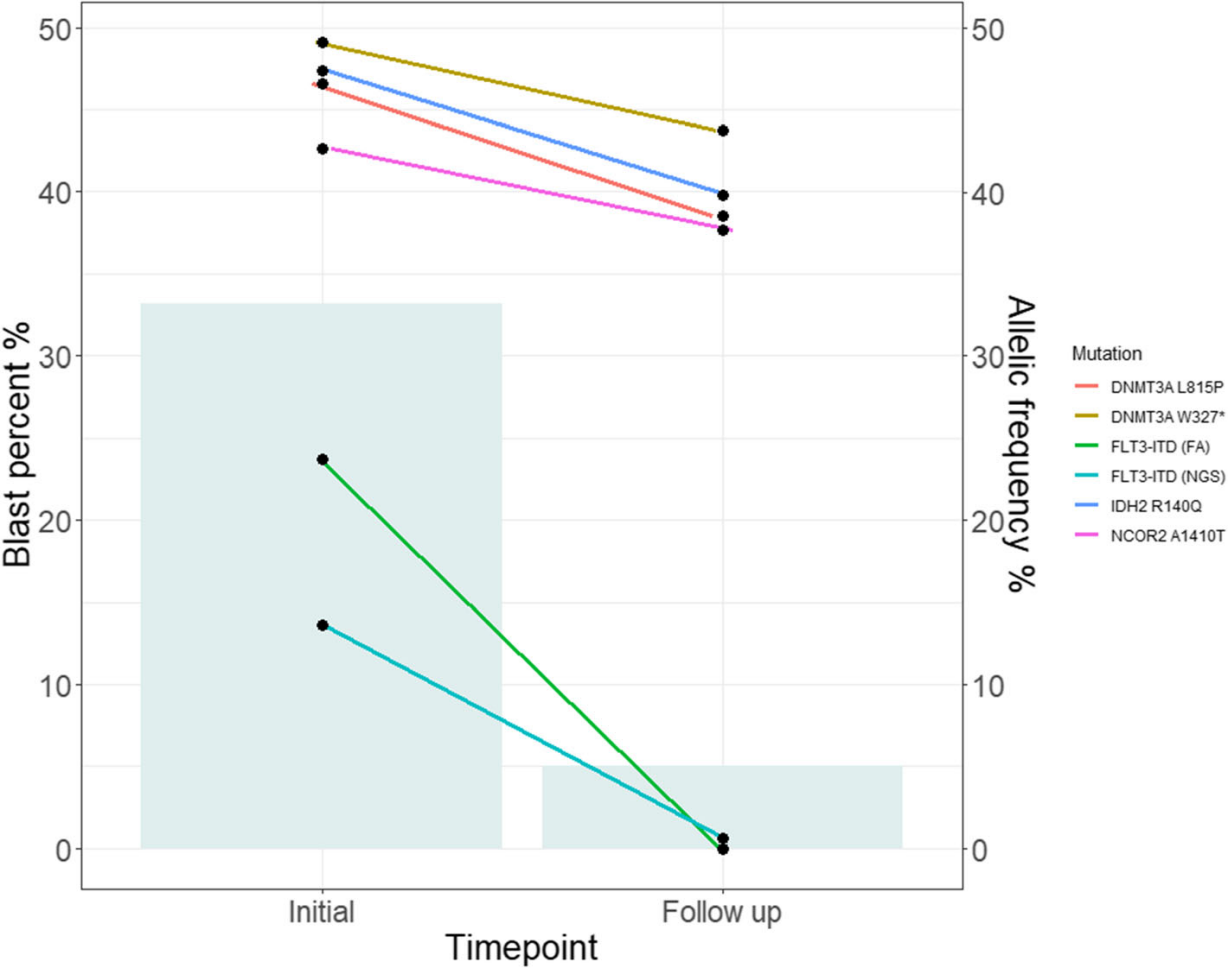
Change in AF values of somatic mutations in an AML case with discordant results from NGS and fragment analysis, respectively (Patient 7). The *FLT3* ITD AR was calculated by fragment analysis was converted into an AF value according to the following equation; AF(%) = AR/(1 − AR)*100. The box represents the blast percentage observed in a bone marrow aspirate smear sample

One AML case (Patient 7) with discordant results from NGS and fragment analysis, respectively, demonstrated a relatively low mutation burden (0.6%) per NGS; this result was obtained from patient’s follow-up sample. A small peak was detected in the fragment analysis from the follow-up sample, but the calculated AR was below the detection limit of fragment analysis that was considered negative. This patient harbored *IDH2* (one missense), *DNMT3A* (one missense and one nonsense), and *NCOR* (one missense) gene mutations together with *FLT3* ITD. After six cycles of hypomethylating therapy, the patient underwent NGS as well as *FLT3* ITD fragment analysis. The mutation burden change of each mutation in this case is depicted in Fig. 3. Mutations other than *FLT3* ITD revealed by fragment analysis persisted in follow-up samples, and bone marrow aspirate showed a residual blast presence of approximately 5%. This patient ultimately relapsed four months into the follow-up period; this outcome suggested that low-level ITD alleles were missed by PCR, yet *FLT3* ITD could be real. NGS presents a better potential to be incorporated into the clinical field in ITD detection. Like in our case, samples with a low mutation burden near the detection limit of the conventional fragment analysis could be detected more by the NGS method.

Quantitative analysis revealed a moderate degree of correlation between two methods in clinical samples (r = 0.757; slope: 0.164). The reason for this low regression slope is thought to be partly due to the well-known tendency of the Pindel algorithm to underestimate the mutant allele fraction [23, 26, 27]. Also, the NGS capture efficiency bias could be the another reason for underestimating the mutation burden in detecting large indels [23, 28]. Accordingly, the ROC curve was analyzed, and, through our results, a cutoff value of 0.11—which is lower than 0.33, a cutoff corresponding to an AR value of 0.5—could be calculated. The diagnostic sensitivity in this case was 85.3%, which was an improved result relative to that achieved when applying the existing criterion to determine the degree of mutation burden. Our result was suggested that the criterion of AF from NGS to determine the degree of mutation burden—which is judged to be related to the clinical prognosis in the current guideline—should be applied differently from 0.33. Future studies should evaluate whether the AF cutoff using NGS has a clinical prognostic value. When using NGS, various factors such as probe design, sequencing chemistry, and bioinformatics pipeline affect the results, so standardization efforts on setting cutoff will also be needed.

Sensitive yet reliable detection of *FLT3* ITD is required in the clinical field, and monitoring of disease status offers a significant advantage for clinicians. Applying NGS analysis could help guide decision-making, including transplantation and timely use of FLT3 inhibitors. Using the NGS method, *FLT3* ITD can be identified sensitively through a single test and other gene mutation patterns, which can increase comprehensive understanding of the disease. In addition, according to the assay and bioinformatics algorithm used, the laboratory should establish a standard for high mutation burden criterion based on established guidelines.

## Supplementary Information

**Additional file 1 Supplementary Table 1**. List of genes included in the hematologic malignancy–target gene panel. **Supplementary Table 2**. Clinical information and *FLT3* ITD results of samples obtained from patients. **Supplementary Table 3**. Clinical and biological characteristics of the studied patients with follow up samples. **Supplementary Fig. 1**. The bioinformatics pipelines used in this study. **Supplementary Fig. 2**. *FLT3* ITD detection result by fragment analysis and by NGS in acute myeloid leukemia patient with discrepant case (Patient 7).
