# Supplementary material for: A comparative study of next-generation sequencing and fragment analysis for the detection and allelic ratio determination of FLT3 internal tandem duplication
Source: Diagn Pathol. 2022 Jan 26;17:14. doi: 10.1186/s13000-022-01202-x (PMC8790841; doi:10.1186/s13000-022-01202-x)
Supplement: Supplementary file 1 — Additional file 1 Supplementary Table 1. List of genes included in the hematologic malignancy–target gene panel. Supplementary Table 2. Clinical information and FLT3 ITD results of samples obtained from patients. Supplementary Table 3. Clinical and biological characteristics of the studied patients with follow up samples. Supplementary Fig. 1. The bioinformatics pipelines used in this study. Supplementary Fig. 2. FLT3 ITD detection result by fragment analysis and by NGS in acute myeloid leukemia patient with discrepant case (Patient 7). [file 13000_2022_1202_MOESM1_ESM.docx]

**Supplementary Table 1.** List of genes included in the hematologic malignancy–target gene panel

| *ABCB1, ABCB7, ABCG2, ABCG5, ABCG8, ABL1, ABL2, ACD, ACTB, ACTN1, ADA, ADAMTS13, AIRE, AK1, AK2, AKT2, ALAS2, ALDOA, AMN, ANK1, ANKRD26, AP3B1, ARID1A, ARPC1B, ASXL1, ATG2B, ATM, ATR, ATRX, AXIN1, BCL11B, BCL2, BCL6, BCOR, BCORL1, BHLHE41, BIRC3, BLM, BPGM, BRAF, BRCA1, BRCA2, BRCC3, BRINP3, BRIP1, BTG1, BTK, BTLA, C3, C4BPA, C4BPB, CALN1, CALR, CARD11, CASP10, CBL, CBLB, CBLC, CCND1, CD200, CD247, CD27, CD36, CD3D, CD3E, CD40LG, CD46, CD58, CD59, CD79B, CDAN1, CDKN1B, CDKN2A, CDKN2B, CEBPA, CFB, CFH, CFHR1, CFHR3, CFHR4, CFHR5, CFI, CHD1, CHD4, CHD9, CHMP2B, CLPB, CNOT3, COX4I2, CREBBP, CRLF2, CSF1R, CSF2RA, CSF3R, CTC1, CTCF, CTSC, CUBN, CUX1, CXCR4, CYB5R3, CYBA, CYBB, CYCS, DCLRE1C, DDX41, DGKE, DGKH, DHFR, DIS3, DKC1, DNM2, DNMT1, DNMT3A, EBF1, ECT2L, EED, EGFR, EGLN1, EGLN2, EGLN3, EHMT1, ELANE, EP300, EPAS1, EPB41, EPB42, EPCAM, EPO, EPOR, ERCC4, ERG, ETNK1, ETV6, EZH2, F2R, FANCA, FANCB, FANCC, FANCD2, FANCE, FANCF, FANCG, FANCI, FANCL, FANCM, FAS, FASLG, FAT1, FBXW7, FCGR1A, FCGR3B, FERMT3, FLI1, FLNA, FLT3, FOXP3, G6PC3, G6PD, GATA1, GATA2, GATA3, GCLC, GFI1, GFI1B, GIF, GINS1, GLRX5, GNAS, GNB1, GP1BA, GP1BB, GP9, GPI, GPRC5A, GPX1, GSKIP, GSN, GSR, GSS, HAX1, HBA1, HBA2, HBB, HBD, HCLS1, HFE, HIF1A, HIF1AN, HIF3A, HK1, HNRNPK, HOOK1, HOXA10, HOXA11, HRAS, HSPA9, HUWE1, ID3, IDH1, IDH2, IFNG, IFNGR1, IFNGR2, IKZF1, IKZF2, IKZF3, IL12RB1, IL2RB, IL2RG, IL3RA, IL7R, IRF1, ITGA2, ITGA2B, ITGB2, ITGB3, ITK, ITPKB, JAGN1, JAK1, JAK2, JAK3, JAKMIP2, JMJD1C, KDM5C, KDM6A, KDM7A, KIF23, KIT, KLF1, KMT2A, KMT2C, KMT2D, KRAS, LAMB4, LAMTOR2, LAPTM5, LCK, LEF1, LIG4, LMNA, LMO1, LMO2, LPIN2, LRP1B, LRRC4, LUC7L2, LYL1, LYST, MAD2L2, MAGT1, MAP2K1, MAP2K2, MASTL, MBL2, MECOM, MED13, MEF2B, MEF2C, MEFV, MET, MLH1, MLLT10, MLLT3, MPL, MSH2, MSH4, MSH6, MTA1, MTAP, MTR, MTRR, MVK, MYB, MYC, MYD88, MYH9, MYSM1, NAF1, NBEAL2, NBN, NCF2, NCOR2, NF1, NHEJ1, NHP2, NLRP3, NOD2, NOP10, NOTCH1, NOTCH2, NPM1, NR3C1, NRAS, NT5C2, NT5C3A, NTRK3, NUP214, OS9, P2RY2, PALB2, PARN, PAX5, PBX1, PC, PCDHB1, PDGFRA, PDGFRB, PDHA1, PDHX, PFKL, PFKM, PGK1, PGM3, PHF6, PICALM, PIEZO1, PIGA, PIK3CD, PIK3R1, PKLR, PML, PMS2, PNP, POT1, PRDM1, PRF1, PRKACG, PRPF40B, PTCH2, PTEN, PTK2B, PTPN11, PTPN2, PTPRC, PTPRD, PUS1, RAB27A, RAC1, RAC2, RAD21, RAD50, RAD51, RAD51C, RAF1, RAG1, RAG2, RB1, RBBP6, RBM8A, RELN, RFWD3, RHAG, RHOA, RIT1, RMRP, RNF168, RPL10, RPL11, RPL15, RPL23, RPL26, RPL27, RPL31, RPL35A, RPL36, RPL5, RPS10, RPS14, RPS15, RPS17, RPS19, RPS24, RPS26, RPS27, RPS27A, RPS28, RPS29, RPS7, RTEL1, RUNX1, RUNX1T1, SAMD9L, SBDS, SBF2, SEC23B, SERPING1, SETBP1, SETD2, SF1, SF3A1, SF3B1, SH2B3, SH2D1A, SHOC2, SLC11A2, SLC19A2, SLC25A38, SLC2A1, SLC35C1, SLC37A4, SLC4A1, SLCO1B1, SLCO1B3, SLFN14, SLX4, SMARCD2, SMC1A, SMC3, SOS1, SPINK5, SPRED1, SPTA1, SPTB, SRC, SRCAP, SRP72, SRSF2, STAG1, STAG2, STAT3, STAT5B, STEAP3, STX11, STXBP2, SUZ12, SYNE1, TAL1, TAL2, TAZ, TBL1XR1, TBX1, TCF3, TCIRG1, TEC, TERC, TERF1, TERF2, TERF2IP, TERT, TET1, TET2, TET3, THBD, THPO, TINF2, TLX1, TLX3, TMPRSS6, TNFAIP3, TNFRSF13B, TNFRSF14, TNFRSF1A, TOX, TP53, TPI1, TPMT, TRAF3, TRNT1, TSLP, TSR2, TUBB1, TYK2, U2AF1, U2AF2, UBE2T, UGT1A1, UGT1A7, UNC13B, UNC13D, UNC5D, USB1, USH2A, USP9X, VHL, VPS13B, VPS45, VWF, WAS, WDR1, WIPF1, WRAP53, WT1, XBP1, XIAP, XK, XRCC2, YARS2, ZAP70, ZFHX4, ZNF197, ZRSR2, MRE11A, WHSC1, STON1, OBFC1* |
| --- |

**Supplementary Table 2.** Clinical information and *FLT3* ITD results of initial samples obtained from patients

| **Patient** | **Age/**  **Gender** | **WBC**  **(/µL)** | **Allelic ratio by FA** | **FLT3-ITD size (Genescan)** | **Allelic ration by NGS** | **FLT3-ITD detection by NGS** | **Insertion site** | ***FLT3* ITD size (NGS with pindel)** | **Diagnosis (blast percentage)** | **Chemotherapy regimen** | **FLT3 inhibitor application** |
| --- | --- | --- | --- | --- | --- | --- | --- | --- | --- | --- | --- |
| 1 | 64/F | 1,350 | 0.08 | 83.90 | 0.031 | c.1734_1817dup | exon 14 | 84 | AML with maturation (23.2%) | Hypomethylating agent | No |
| 2 | 39/F | 28,950 | 1.48 | 89.71 | 0.112 | c.1749_1837+1dup | intron 14 | 90 | AML with mutated *NPM1* (72.9%) | Induction chemotherapy | Yes |
| 3 | 15/M | 149,800 | 0.15 | 32.67 | 0.147 | c.1798_17995ins48 | exon 14 | 48 | AML with biallelic mutation of *CEBPA* (76.0%) | Induction chemotherapy | No |
| 4 | 60/F | 207,200 | 28.34 | 21.00 | 0.937 | c.1784_1804dup | exon 14 | 21 | AML with mutated *NPM1* (96.0%) | Induction chemotherapy | No |
| 5 | 15/M | 1,960 | 0.31 | 45.14 | 0.164 | c.1761_1805dup | exon 14 | 45 | AML with myelodysplasia-related change (49.0%) | Induction chemotherapy | No |
| 6 | 45/F | 54,430 | 3.17 | 53.77 | 0.374 | c.1740_1793dup | exon 14 | 54 | AML with myelodysplasia-related change (22.7%) | Induction chemotherapy | No |
| 7 | 76/F | 57,200 | 0.31 | 92.88 | 0.136 | c.1807_1899dup | exon 14 | 93 | AML with maturation (33.2%) | Hypomethylating agent | No |
| 8 | 50/F | 1,400 | 0.64 | 50.98 | 0.143 | c.1831_1832ins51 | exon 14 | 51 | AMMoL (80.1%) | Induction chemotherapy | Yes |
| 9 | 49/F | 125,330 | 0.58 | 171.89 | 0.191 | c.1787_1879dup | exon 15 | 183 | AML with mutated *NPM1* (95.7%) | Induction chemotherapy | No |
| 10 | 53/M | 102,300 | 7.37 | 137.88 | 0.103 | c.1870_1871ins138 | exon 15 | 138 | AML with maturation (63.0%) | Induction chemotherapy | No |
| 11 | 76/M | 122,500 | 0.73 | 41.86 | 0.206 | c.1796_1837dup | exon 14 | 42 | AML with mutated *NPM1* (88.3%) | Hypomethylating agent | No |
| 12 | 71/M | 3,990 | 0.46 | 68.74 | 0.112 | c.1766_1834dup | exon 14 | 69 | AML with maturation (75.0%) | Hypomethylating agent | No |
| 13 | 82/F | 93,540 | 0.16 | 60.01 | 0.023 | c.1805_1806ins60 | exon 14 | 60 | AMoL (51.1%) | Hypomethylating agent | No |
| 14 | 52/F | 3,870 | 1.17 | 104.74 | 0.133 | c.1837+20_1837+21ins105 | intron 14 | 105 | AML with maturation (72.1%) | Hypomethylating agent | No |
| 15 | 46/M | 17,310 | 0.86 | 47.55 | 0.254 | c.1734_1781dup | exon 14 | 48 | AML with mutated *NPM1* (54.0%) | Induction chemotherapy | No |
| 16 | 64/M | 93,560 | 0.16 | 166.31 | 0.010 | c.1838-2_1838-1ins167 | intron 14 | 167 | AML with mutated *NPM1* (89.3%) | Hypomethylating agent | No |
| 17 | 39/M | 14,840 | 0.70 | 18.31 | 0.201 | c.1770_1787dup | exon 14 | 18 | AML with myelodysplasia-related change (55.6%) | Induction chemotherapy | No |
| 18 | 24/M | 144,800 | 0.36 | 63.47 | 0.193 | c.1772_1834dup | exon 14 | 63 | AML with biallelic mutation of *CEBPA* (89.2%) | Induction chemotherapy | No |
| 19 | 83/M | 97,270 | 0.02 | 30.18 | 0.018 | c.1805_1806ins30 | exon 14 | 30 | AML with mutated *NPM1* (84.1%) | Hypomethylating agent | No |
| 20 | 40/F | 27,350 | 0.85 | 29.66 | 0.288 | c.1767_1796dup | exon 14 | 30 | AML with maturation (64.4%) | Hypomethylating agent | No |
| 21 | 46/M | 206,260 | 0.22 | 38.83 | 0.034 | c.1812_1813ins39 | exon 14 | 39 | AML with biallelic mutation of *CEBPA* (85.4%) | Induction chemotherapy | No |
| 22 | 86/M | 148,600 | 0.18 | 54.15 | 0.101 | c.1788_1832dup | exon 14 | 45 | AML with mutated *NPM1* (91.5%) | Hypomethylating agent | No |
| 23 | 54/F | 186,880 | 2.08 | 66.02 | 0.538 | c.1837_1837+1ins66 | intron 14 | 66 | AML with mutated *NPM1* (81.3%) | Induction chemotherapy | Yes |
| 24 | 36/M | 255,200 | 0.56 | 39.27 | 0.234 | c.1782_1820dup | exon 14 | 39 | AML with maturation (69.6%) | Induction chemotherapy | Yes |
| 25 | 70/F | 13,180 | 0.09 | 56.90 | 0.045 | c.1748_1804dup | exon 14 | 57 | AML with mutated *NPM1* (44.7%) | Hypomethylating agent | No |
| 26 | 34/F | 94,960 | 0.76 | 38.88 | 0.226 | c.1770_1771ins39 | exon 14 | 39 | AML with t(8;21)(q22;q22.1); *RUNX1-RUNX1T1* (64.9%) | Induction chemotherapy | No |
| 27 | 63/F | 22,130 | 0.16 | 158.01 | 0.016 | c.1857_1858ins159 | exon 15 | 159 | AMMoL (53.8%) | Induction chemotherapy | Yes |
| 28 | 65/M | 21,250 | 0.18 | 41.69 | 0.088 | c.1797_1798ins42 | exon 14 | 42 | AML with maturation (45.5%) | Hypomethylating agent | No |
| 29 | 12/M | 2,350 | 0.62 | 53.82 | 0.297 | c.1740_1793dup | exon 14 | 54 | AML with myelodysplasia-related change (55.1%) | Induction chemotherapy | No |
| 30 | 48/F | 55,440 | 0.54 | 24.72 | 0.153 | c.1777_1800dup | exon 14 | 24 | AML with mutated *NPM1* (73.5%) | Induction chemotherapy | No |
| 31 | 9/F | 1,330 | 0.64 | 50.68 | 0.266 | c.1742_1792dup | exon 14 | 51 | APL (57.9%) | Vesanoid + idarubicin | No |
| 32 | 64/F | 143,200 | 0.69 | 53.91 | 0.123 | c.1773_1826dup | exon 14 | 54 | AML with mutated *NPM1* (90.8%) | Induction chemotherapy | Yes |
| 33 | 55/M | 235,080 | 0.70 | 39.64 | 0.168 | c.1748_1786dup | exon 14 | 39 | AML with mutated *NPM1*(92.3%) | Induction chemotherapy | No |
| 34 | 58/M | 1,820 | 0.13 | 56.10 | 0.045 | c.1745_1804dup | exon 14 | 60 | AML with maturation (38.8%) | Induction chemotherapy | Yes |
| 35 | 63/M | 7,860 | 0.02 | 47.80 | 0.005 | c.1837+1_1837+2ins48 | intron 14 | 48 | AML with mutated *NPM1* (21.0%) | - | - |
| 36 | 38/M | 5,210 | 0.84 | 20.53 | 0.293 | c.1784_1804dup | exon 14 | 21 | AML with maturation (90.1%) | Induction chemotherapy | Yes |
| 37 | 15/F | 143,900 | 0.36 | 69.06 | 0.091 | c.1766_1834dup | exon 14 | 69 | AML with maturation (41.1%) | Induction chemotherapy | No |
| 38 | 55/M | 139,500 | 0.40 | 83.61 | 0.066 | c.1837_1837+1ins84 | intron 14 | 84 | AML with mutated *NPM1* (93.6%) | Induction chemotherapy | No |
| 39 | 57/F | 30,560 | 0.28 | 62.92 | 0.031 | c.1780_1781ins63 | exon 14 | 63 | AML with mutated *NPM1* (51.9%) | Induction chemotherapy | Yes |
| 40 | 65/F | 155,600 | 1.49 | 181.60 | 0.069 | c.1787_1879dup | exon 15 | 183 | AML with mutated *NPM1* (86.3%) | Hypomethylating agent | No |
| 41 | 23/M | 76,270 | 0.72 | 39.07 | 0.131 | c.1786_1824dup | exon 14 | 39 | AML with mutated *RUNX1* (70.7%) | Induction chemotherapy | Yes |
| 42 | 34/M | 376,400 | 0.75 | 39.33 | 0.047 | c.1818_1819ins39 | exon 14 | 39 | AMMoL (84.6%) | Induction chemotherapy | Yes |
| 43 | 74/M | 6,670 | 1.95 | 38.95 | 0.257 | c.1737_1775dup | exon 14 | 39 | AMoL (52.4%) | Hypomethylating agent | Yes |
| 44 | 67/F | 3,010 | 0.19 | 50.91 | 0.031 | c.1744_1794dup | exon 14 | 51 | AML with mutated *NPM1* (39.2%) | Hypomethylating agent | No |
| 45 | 72/M | 1,820 | 0.51 | 24.04 | 0.224 | c.1795_1796ins24 | exon 14 | 24 | AML with mutated *NPM1* (75.0%) | Hypomethylating agent | No |
| 46 | 24/M | 5,400 | 2.83 | 24.02 | 0.497 | c.1777_1800dup | exon 14 | 24 | AML with mutated *NPM1* (96.8%) | Induction chemotherapy | No |
| 47 | 72/M | 8,230 | 0.31 | 29.64 | 0.109 | c.1789_1790ins30 | exon 14 | 30 | AML with mutated *RUNX1* (85.0%) | Hypomethylating agent | No |
| 48 | 77/F | 25,280 | 0.36 | 214.54 | 0.078 | c.1745_1870dup | exon 15 | 216 | AML with mutated *NPM1* (79.2%) | Hypomethylating agent | Yes |
| 49 | 70/F | 10,110 | 0.44 | 80.62 | 0.018 | c.1838_1839ins81 | exon 15 | 81 | AMMoL (92.1%) | Hypomethylating agent | No |
| 50 | 83/M | 8,030 | 0.31 | 53.73 | 0.079 | c.1793_1794ins54 | exon 14 | 54 | APL (75.8%) | Vesanoid | No |
| 51 | 77/M | 120,680 | 0.04 | 56.87 | 0.006 | c.1747_1803dup | exon 14 | 57 | AML with mutated *NPM1* (94.6%) | Induction chemotherapy | Yes |
| 52 | 58/F | 2,490 | 0.26 | 27.06 | 0.052 | c.1796_1797ins27 | exon 14 | 27 | AML with myelodysplasia-related change (31.2%) | Induction chemotherapy | Yes |
| 53 | 69/F | 114,150 | 3.14 | 164.93 | 0.245 | c.1838_1839ins165 | exon 15 | 165 | AML with maturation (85.1%) | Hypomethylating agent | No |
| 54 | 59/F | 5,350 | 0.10 | 92.62 | 0.008 | c.1838_1839ins93 | exon 15 | 93 | AML with maturation (28.2%) | Induction chemotherapy | No |
| 55 | 78/M | 43,390 | 0.54 | 41.62 | 0.139 | c.1763_1764ins42 | exon 14 | 42 | AMMoL (74.8%) | Hypomethylating agent | No |
| 56 | 41/M | 1,690 | 0.05 | 23.94 | 0.012 | c.1760_1783dup | exon 14 | 24 | APL (72.9%) | Vesanoid | No |
| 57 | 41/F | 17,790 | 0.32 | 42.16 | 0.075 | c.1815_1816ins24 | exon 14 | 42 | AML wit t(8;21)(q22;q22.1); *RUNX1-RUNX1T1* (29.2%) | Induction chemotherapy | Yes |
| 58 | 82/M | 11,820 | 0.02 | 86.71 | 0.010 | c.1837+33_1837+34ins87 | intron 14 | 87 | AML with inv(16)(p13.1q22) (18.2%) | Hypomethylating agent | No |
| 59 | 22/F | 21,450 | 0.66 | 68.78 | 0.146 | c.1800_1801ins69 | exon 14 | 69 | AML with maturation (70.9%) | Induction chemotherapy | Yes |
| 60 | 59/M | 41,960 | 2.79 | 140.06 | 0.170 | c.1793_1843dup | exon 15 | 141 | AML with mutated *NPM1* (90.3%) | Induction chemotherapy | Yes |
| 61 | 40/M | 3,010 | 0.73 | 47.83 | 0.147 | c.1747_1794dup | exon 14 | 48 | AML with mutated *NPM1* (85.3%) | Induction chemotherapy | Yes |

Abbreviations: FA, fragment analysis; NGS, next-generation sequencing; ITD, internal tandem duplication; AML, acute myeloid leukemia; APL, acute promyelocytic leukemia; AMMoL, acute myelomonocytic leukemia; AMoL, acute monocytic leukemia

**Supplementary Table 3.** Clinical and biological characteristics of the studied patients with follow up samples

| **Patient** | **Status** | **F/U month** | **Age/Gender** | **Allelic ratio by FA** | ***FLT3* ITD size (Genescan)** | **Allelic ration by NGS** | ***FLT3* ITD size (NGS with pindel)** | **WBC count (/µL)** | **Diagnosis** | **Response to chemotherapy** | **ASCT** | **Relapse** | **Death** |
| --- | --- | --- | --- | --- | --- | --- | --- | --- | --- | --- | --- | --- | --- |
| 1 | Initial | - | 64/F | 0.08 | 83.9 | 0.031 | 84 | 1,350 | AML with maturation | CR | No | No | Yes |
|  | Follow up #1 | 2 |  | ND | - | ND | - | 740 |  |  |  |  |  |
|  | Follow up #2 | 4 |  | ND | - | ND | - | 1,410 |  |  |  |  |  |
| 2 | Initial | - | 39/F | 1.48 | 89.71 | 0.112 | 90 | 28,950 | AML with mutated *NPM1* | Failure | Yes | Yes | No |
|  | Follow up #1 | 2 |  | 0.62 | 89.73 | 0.104 | 90 | 2,130 |  |  |  |  |  |
| 3 | Initial | - | 15/M | 0.15 | 32.67 | 0.147 | 48 | 149,800 | AML with biallelic mutation of *CEBPA* | CR | Yes | No | No |
|  | Follow up #1 | 2 |  | ND | - | ND | - | 1,310 |  |  |  |  |  |
|  | Follow up #2 | 12 |  | ND | - | ND | - | 5,640 |  |  |  |  |  |
|  | Follow up #3 | 14 |  | ND | - | ND | - | 7,900 |  |  |  |  |  |
| 4 | Initial | - | 60/F | 28.34 | 21 | 0.937 | 21 | 207,200 | AML with mutated *NPM1* | Failure | Yes | Yes | Yes |
|  | Follow up #1 | 12 |  | 12.56 | 21 | 0.778 | 21 | 26,670 |  |  |  |  |  |
| 5 | Initial | - | 15/M | 0.31 | 45.14 | 0.164 | 45 | 1,960 | AML with myelodysplasia-related change | CR | Yes | No | No |
|  | Follow up #1 | 1 |  | ND | - | ND | - | 290 |  |  |  |  |  |
|  | Follow up #2 | 3 |  | ND | - | ND | - | 220 |  |  |  |  |  |
|  | Follow up #3 | 7 |  | ND | - | ND | - | 3,300 |  |  |  |  |  |
|  | Follow up #4 | 12 |  | ND | - | ND | - | 4,170 |  |  |  |  |  |
|  | Follow up #5 | 18 |  | ND | - | ND | - | 7,310 |  |  |  |  |  |
|  | Follow up #6 | 31 |  | ND | - | ND | - | 5,890 |  |  |  |  |  |
| 6 | Initial | - | 45/F | 3.17 | 53.77 | 0.374 | 54 | 54,430 | AML with myelodysplasia-related change | PR | Yes | No | Yes |
|  | Follow up #1 | 2 |  | ND | - | ND | - | 3,480 |  |  |  |  |  |
| 7 | Initial | - | 76/F | 0.31 | 92.88 | 0.136 | 93 | 57,200 | AML with maturation | Failure | No | Yes | Yes |
|  | Follow up #1 | 2 |  | ND | - | 0.006 | 93 | 2,390 |  |  |  |  |  |
| 8 | Initial | - | 50/F | 0.64 | 50.98 | 0.143 | 51 | 1,400 | AMMoL | Failure | Yes | Yes | Yes |
|  | Follow up #1 | 5 |  | 1.14 | 50.98 | 0.155 | 51 | 14,250 |  |  |  |  |  |

Abbreviations: FA, fragment analysis; NGS, next-generation sequencing; ITD, internal tandem duplication; ND, not detected; AML, acute myeloid leukemia; CR, complete remission; PR, partial remission; AMMoL, acute myelomonocytic leukemia; AMoL, acute monocytic leukemia; ASCT, allogeneic hematopoietic stem cell transplantation.

**Supplementary Figure 1.** The bioinformatics pipelines used in this study


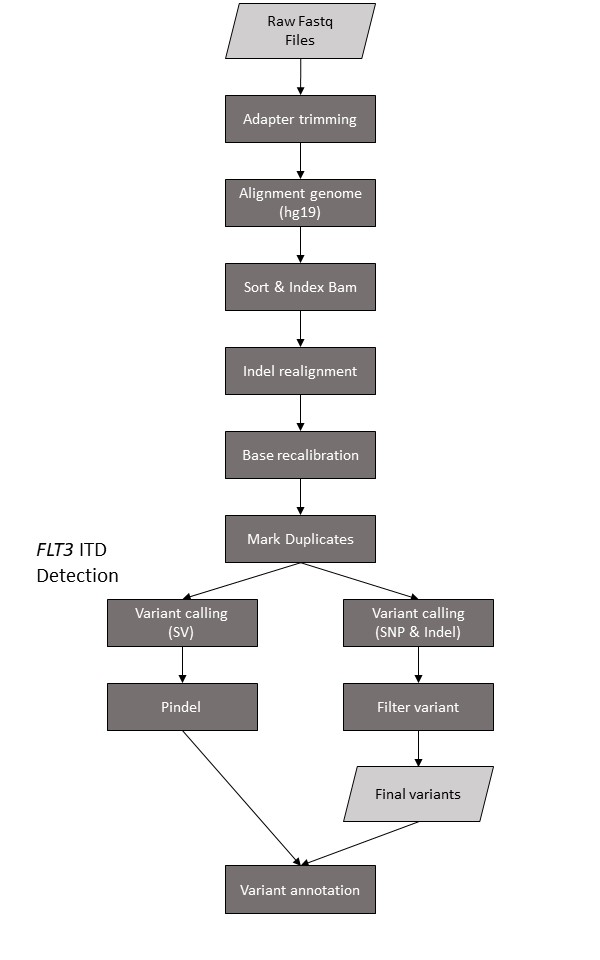


**Supplementary Figure 2.** *FLT3* ITD detection result by fragment analysis and by NGS in acute myeloid leukemia patient with discrepant case (Patient 7) (A) Electropherogram display *FLT3* amplicon signals as fragment peaks with the distance of the peak positions corresponding to the size of the ITD and the peak height used for calculation of the mutational burden; the x-axis represents the PCR product size in base pair (bp), and the y-axis represents the fluorescence intensity. The *FLT3* ITD/wild-type allelic ratio was 0.31 for the initial sample. (B) NGS read alignment displayed using the Integrative Genomics Viewer (Broad Institute, Cambridge, MA, USA), demonstrating the presence of an ITD (c.1807_1899dup; p.S633_K634ins31) with allele fraction (*FLT3* ITD/total) of 13.6% and a corresponding allelic ratio (*FLT3* ITD/wild type) of 0.157 *FLT3* ITD showed only partial alignment with the reference sequence. (C) Fragment analysis of follow-up of a sample of (A) showed only wild-type peak while the (D) NGS displayed the presence of an ITD in low mutation burden, the allelic fraction of 0.6%.

Abbreviations: WT, wild type

**
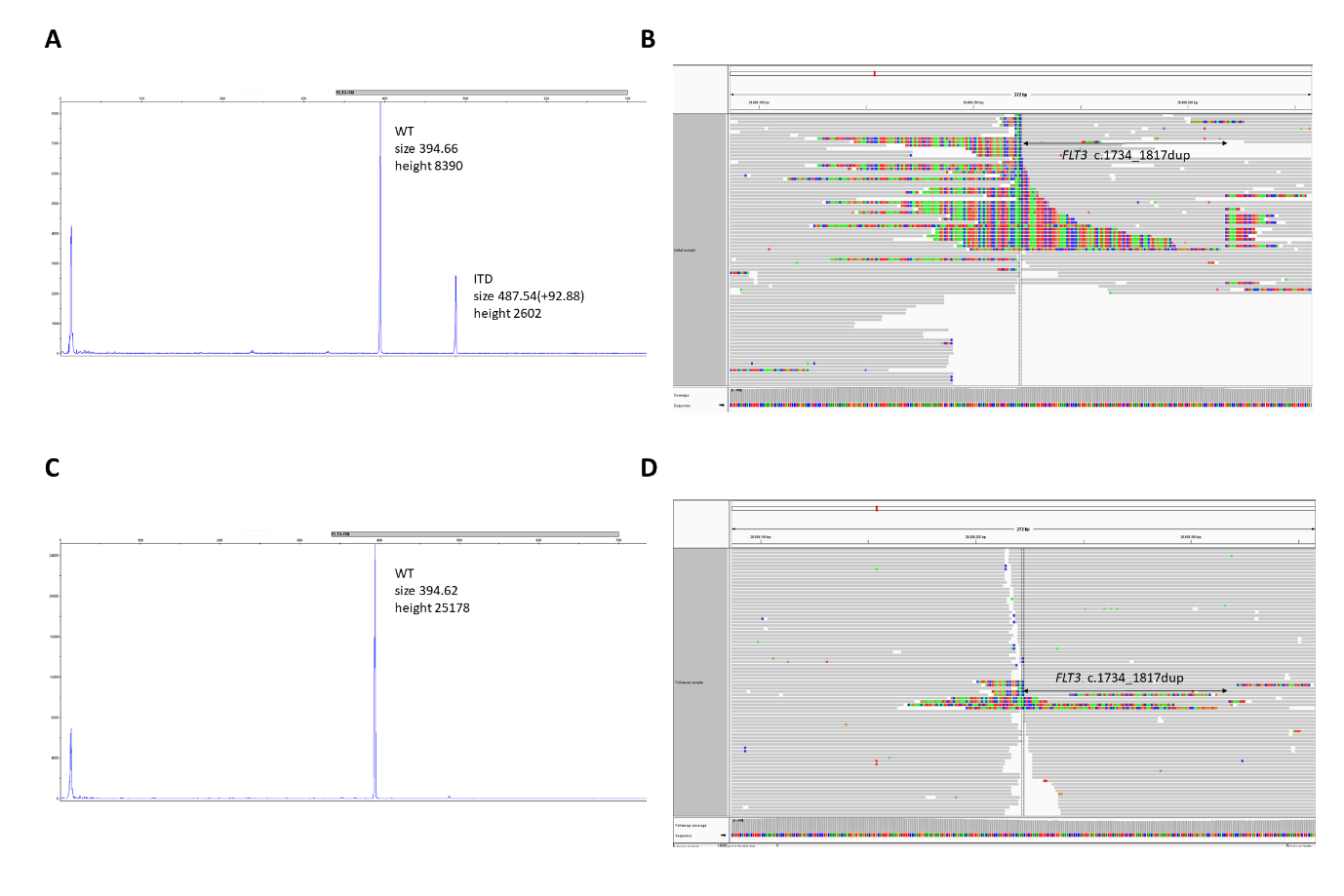
**
